# 697. Characteristics of Hospitalized Patients Colonized with *Clostridioides difficile* at an Academic Medical Center

**DOI:** 10.1093/ofid/ofad500.759

**Published:** 2023-11-27

**Authors:** Kelly M Percival, Patrick M Kinn, Bradley A Ford, Karen Brust, Dilek Ince

**Affiliations:** University of Iowa Hospitals & Clinics, Iowa City, Iowa; University of Iowa Hospitals & Clinics, Iowa City, Iowa; University of Iowa Hospitals and Clinics, Iowa City, Iowa; University of Iowa Hospitals & Clinics, Iowa City, Iowa; University of Iowa Hospitals & Clinics, Iowa City, Iowa

## Abstract

**Background:**

Clinical spectrum of *C. difficile* can range from colonization to fulminant colitis. Patients with toxin antigen negative, *C. difficile* nucleic acid amplification (NAAT)-positive diarrhea are frequently only colonized and do not suffer consequences of *C. difficile* associated disease (CDAD) but receive treatment for CDAD due to the perceived low risk of oral vancomycin vs. the risk of developing CDAD.

**Methods:**

We performed a retrospective cohort study of hospitalized adult patients with positive *C. difficile* NAAT and negative toxin antigen results at University of Iowa Health Care between 9/1/2021 and 9/30/2022. Chart review was conducted to obtain patient age, immunocompromised state, involvement of infectious disease (ID) consultant, prior *C. difficile* test status and treatment course.

**Results:**

Of the 295 NAAT-positive *C. difficile* results, 210 (71%) were toxin antigen negative. Patient characteristics and frequency of common risk factors are shown in Table 1. *C. difficile* colonization was detected ≥4 days after admission in 111 (53%), with internal medicine and stem cell transplant (SCT)/hematologic malignancies (HM) services having the most patients with colonization (31% and 18%, respectively). Antibiotic use in the prior 30 days was reported in 154 (73%). Of 134 (64%) patients on systemic antibiotics at time of *C. difficile* testing, antibiotics were discontinued in 29 (22%). Only 43 patients (20%) did not receive any treatment for *C. difficile*. Median treatment duration was 10 days with 133 (80%) being treated for 10-14 days (Table 2). ID consult was obtained in 48 (23%) and recommended no treatment initially in 11 (23%), 4 of whom were started on treatment in subsequent days. Oral twice a day vancomycin for prophylaxis was given to 28 (13%) patients, 19 (68%) of whom were immunocompromised (16 SCT/HM and 3 solid organ transplant).

**Figure:**
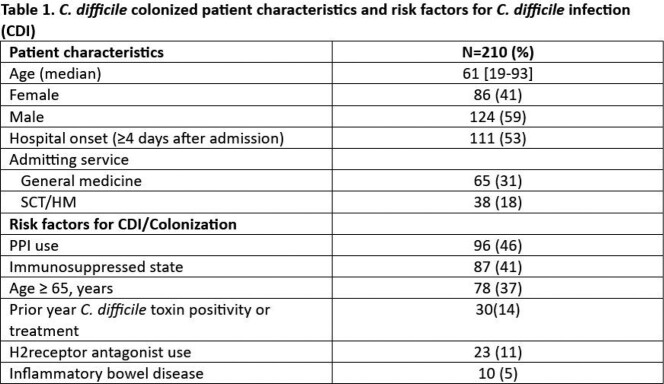

**Figure:**
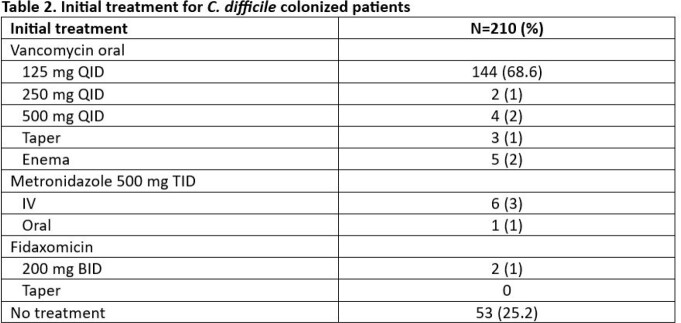

**Conclusion:**

Patients with *C. difficile* colonization have similar risk factors as patients with CDAD. Understanding treatment patterns for *C. difficile* colonization can guide educational activities. Most colonized patients received *C. difficile* treatment and/or prophylaxis, likely due to ongoing systemic antibiotic use and immunosuppression. Benefit of treatment and prophylaxis in colonized patients remains an unresolved issue.

**Disclosures:**

**Kelly M. Percival, PharmD**, Gilead Sciences Inc: Advisor/Consultant

